# The oral and maxillofacial surgery training pathway in India

**DOI:** 10.1308/rcsann.2025.0097

**Published:** 2025-11-26

**Authors:** V Sahni

**Affiliations:** All India Institute of Medical Sciences, New Delhi, India

I write further to a recent paper in *The Annals* entitled ‘A comparison of general surgery training programmes across 11 countries: improving understanding of the experience level of international medical graduates in the UK’.^1^

In the context of India, the surgical speciality of oral and maxillofacial surgery (OMFS) deserves special mention, particularly in light of the difference in training pathways between India and the United Kingdom.

In India, OMFS is a dental specialisation. It requires the candidate to have completed a Bachelor of Dental Surgery (BDS) degree at the undergraduate level. Entry to the BDS course is via a national-level entrance examination which is multiple choice-based. The course itself is 5 years in duration, and includes 1 year of compulsory rotatory internship. Completion of the BDS course makes one eligible to sit the national-level entrance examination for the Master in Dental Surgery (MDS), which is again a multiple choice-based exam. OMFS is one of the dental specialities candidates can opt for at the centre of their choice, and is allotted based on availability as per their score and national rank.

The MDS OMFS course is 3 years in duration. It requires the maintenance of a logbook and has a research dissertation component. The exit examination has both a theory and practical component. Both the BDS and MDS degrees are registrable qualifications in India. The Dental Council of India provides procedural guidelines that include intramuscular and intravenous injections, minor surgical procedures, surgical exodontia, removal of impacted dentition, pre-prosthetic procedures, maxillofacial trauma, maxillofacial infections, benign tumours, salivary gland calculi, implants, orthognathic surgery, harvesting cartilage and bone grafts, temporomandibular joint surgery, oncosurgery, jaw resections, cleft lip and palate, emergency management, rhinoplasty, microvascular anastomosis, distraction osteogenesis, skull base surgery and access osteotomies (this list is not exhaustive but attempts to convey the scope of training).^2^

A number of fellowship courses exist in India covering cleft lip and palate, oncosurgery, trauma and orthognathic surgery; however, these courses are not regulated by the Dental Council of India.

The post-qualification pathway for OMFS varies depending upon which sector of healthcare is being talked about. In private non-teaching hospitals and clinics, most post-MDS clinicians are labelled consultants. In private dental colleges, most entry-level nomenclature is that of a senior lecturer or assistant professor. In government dental colleges, the entry-level nomenclature is a senior resident (entry is by means of either an interview or written examination or both).

## Competing interests

The author/s declare no competing interests

## Funding

The author received no financial support for the research, authorship, and/or publication of this article.

## Author contributions

**V Sahni**: Conceptualization, Data curation, Formal analysis, Investigation, Methodology, Project administration, Resources, Software, Supervision, Validation, Visualization, Writing – original draft, Writing – review & editing.

## Artificial intelligence

The author/s declare that no AI was used to conduct the study or prepare the manuscript.
